# Diabetologists’ Knowledge and Prescription of Physical Activity in Southeast Europe

**DOI:** 10.3390/medicina61071244

**Published:** 2025-07-10

**Authors:** Krešimir Martinac, Spomenka Ljubić, Dario Rahelić, Tomas Matić, Tomislav Perković, Slavica Sović

**Affiliations:** 1 Vuk Vrhovac University Diabetes Clinic, Merkur Clinical Hospital, Dugi dol 4a, 10000 Zagreb, Croatiadario.rahelic@gmail.com (D.R.); tomislavperkovic95@gmail.com (T.P.); 2 School of Medicine, University of Zagreb, Šalata 2, 10000 Zagreb, Croatia; 3 School of Medicine, Catholic University of Croatia, Ilica 244, 10000 Zagreb, Croatia; 4 Faculty of Medicine, University Josip Juraj Strossmayer Osijek, Josipa Huttlera 4, 31000 Osijek, Croatia; 5 Andrija Štampar School of Public Health, Johna Davidsona Rockfellera 4, 10000 Zagreb, Croatia

**Keywords:** diabetes, physical activity, counselling

## Abstract

*Background and Objectives*: Physical activity represents a cornerstone in the management of diabetes mellitus; however, the majority of persons with diabetes remain insufficiently active. Current guidelines emphasise the need to educate individuals with diabetes on physical activity at each clinical visit. The aim of the study was to develop an adequate instrument and test diabetologists’ knowledge on physical activity and related guidelines in diabetes, as well as investigate their prescribing habits. *Materials and Methods*: The instrument was developed with four diabetologists using Delphi and RAND appropriateness methods, then distributed to diabetologist societies in 12 Southeast European countries for anonymous completion by their members. Data were analysed using Mann–Whitney U test and Pearson ${\;x}^{2}$ test. *Results*: Out of 302 analysed respondents, 123 (41%) worked at university hospitals, at average four days a week in outpatient departments, mean number of patients was 16 a day; 95 (32%) had a PhD, and 105 (35%) university teaching positions. Median overall knowledge was 15 (Q1–Q3, 13–17) out of 22 maximum points. 26% diabetologists knew recommended level of physical activity for people with diabetes. Physical activity goals were discussed during check-ups in 53.0% of cases; re-education and goal adjustments were provided in 33.4% when health status changed; and clear instructions were documented in 25.5% of medical records. *Conclusions*: Diabetologists’ overall knowledge of physical activity was found to be at least satisfactory in 90% of participants. Knowledge of the guidelines is insufficient in 75% of participants. The prescribing habits are inadequate in half of the participants.

## 1. Introduction

In spite of the development of pharmacological solutions, physical activity and diabetic diet remain the cornerstones of diabetes management owing to their effectiveness. It is precisely the sedentary lifestyle together with changes in eating habits and calorie-rich food that have led to an increase in body weight of the present-day man and increased the prevalence of diabetes. Today, there are 463 million people with diabetes in the world, and the prevalence is constantly increasing [1]. The increase in the number of patients as well as the development of new drugs lead to a constant rise in the cost of diabetes treatment, but at the same time a large number of patients do not achieve a satisfactory level of HbA1c despite pharmacotherapy [2]. On the other hand, physical activity itself can reduce HbA1c by up to one percent [3,4].

Aerobic physical activity includes longer-term physical activity of lower or moderate intensity. Anaerobic physical activity implies high muscle strain that lasts a short time (from several seconds to one minute), during which the muscles do not use oxygen for the rapid creation of adenosine triphosphate (ATP). Both aerobic and anaerobic physical activities have an important effect on glycaemic control, reducing the value of HbA1c by an average of 0.5–0.7% [5,6,7,8]. More exercise leads to a greater reduction in HbA1c independent of the weight loss. Continuous glucose monitoring (CGM) studies have shown that people who exercise regularly have a higher percentage of time spent within target glucose range and less postprandial glycaemic spikes [9]. Regular physical activity, both aerobic and anaerobic, leads to changes in skeletal muscles, adipose tissue, liver and oxidative capacity that increase insulin sensitivity of tissues [10,11,12,13]. Not only does it significantly influence the development of chronic complications of diabetes [14,15,16], but it also exerts a beneficial effect on other components of the metabolic syndrome, such as blood pressure, lipid profile and body weight, and significantly reduces cardiovascular risk [11,17,18,19,20,21].

Although each type of physical activity has a significant effect on glycaemia, research has shown optimal effects in people with diabetes have been achieved by a combination of aerobic and anaerobic exercises [3,8,22,23]. Therefore, current recommendations for persons with both types of diabetes mellitus include engagement in at least 150 min of moderate-intensity aerobic exercise a week and, in the absence of contraindications, encouragement to performing resistance training at least twice a week [17].

In spite of the benefits of physical activity, the majority of persons with diabetes mellitus remain physically inactive and do not follow recommendations [24,25]. Hence, increasing the number of persons with diabetes mellitus who engage in regular physical activity is essential. Various strategies have been proposed, and among them physician-based counselling and education showed good results [26,27,28,29,30]. Physician-based counselling for physical activity should therefore be provided at every patient visit to the physician’s office, as the joint guidelines for the treatment of diabetes of the American Diabetes Association (ADA) and the European Association for the Study of Diabetes (EASD) clearly recommend [31]. However, studies carried out so far have shown that exercise-related advice or referral occurred at no more than half of office visits, and that physicians might lack knowledge of current recommendations on the level of physical activity [28,32,33]. Also, previous studies have assessed physicians’ knowledge of physical activity primarily through their familiarity with guidelines on its implementation. Mere knowledge of the guidelines is not a sufficient element for the effective prescription of physical activity; adequate understanding of the characteristics of both aerobic and anaerobic physical activity is also required. A more detailed analysis of physicians’ knowledge of aerobic and anaerobic physical activity has not yet been conducted. Among the large number of healthcare providers involved in the process of treating persons with diabetes mellitus, diabetologists play the pivotal role. It is therefore very important that they have good knowledge of both types of physical activity and current recommendations for physical activity in persons with diabetes in order to prescribe them correctly and on a regular basis to each and every one of their patients with the aim of promoting involvement of persons with diabetes in regular physical activity.

Since diabetologists’ knowledge on physical activity, its importance and current recommendations, as well as prescribing habits have not been evaluated so far, this study was undertaken to investigate this important topic.

## 2. Materials and Methods

### 2.1. Instrument Development

Ethical approval for the study was obtained from the School of Medicine of the University of Zagreb, Zagreb, Croatia, and the Vuk Vrhovac University Diabetes Clinic, Merkur Clinical Hospital, Zagreb, Croatia. All nurse educators and patients who participated in the focus groups, as well as the experts involved in the development of the instrument, signed informed consent to study participation. The first phase of the research was the development of the instrument according to the principles of the Delphi and the RAND appropriateness methods [34,35]. The questions for the instrument were formulated based on two sources: the guidelines of the American and European Societies for Diabetes, current at the time of the research, and relevant scientific and professional literature obtained from the bibliographic searches of relevant databases—PubMed, Web of Science and Scopus, using the following keywords: diabetes, physical activity, continuing education and instrument. The instrument contained four thematic units: 1. the doctor’s knowledge about physical activity, 2. the doctor’s knowledge about the connection between physical activity and diabetes, 3. knowledge of current guidelines on the type of physical activity and the way it is prescribed to people with diabetes, and 4. the frequency and pattern of prescribing physical activity to people with diabetes as part of diabetologists’ daily routine. In this phase of instrument development, focus groups were organised with diabetes nurse educators and persons with diabetes mellitus. The topics in the focus group of nurse educators included assessment of education on the effect of physical activity on diabetes provided to people with diabetes and description of the implementation of education while identifying its advantages and disadvantages; whereas, in the group of people treated for diabetes, the focus was on whether the doctor informed them about the method, time and type of physical activity as part of the treatment, and what recommendations, in their opinion, best fostered a change in their attitude towards engagement in physical activity. The analysis of the guidelines, the texts obtained by literature database searches, and the analysis of the focus groups resulted in the formation of an instrument containing 50 questions. The instrument was then delivered to four experts in the field of diabetes care, one of whom is also an expert in the field of kinesiology and a lecturer at the Faculty of Kinesiology, University of Zagreb, Zagreb, Croatia. In addition to the evaluation of the proposed questions, the experts were asked to, if necessary, suggest additional questions, and recognise elements of care that had not been covered by the proposed questions. Communication was performed by e-mail according to the principles of the Delphi method. After each expert analysis, a new instrument was created and sent again to the experts for further analysis until they agreed on the final instrument. Before finalising the instrument, a formal meeting with experts was also held. The result of this phase was an instrument that consisted of multiple-choice questions and questions for evaluation on a Likert-type scale, as well as data on age, gender, place of work, daily work routine, academic degree and attendance at diabetes congresses. The instrument is shown in the Appendix A. Scoring of the knowledge section was conducted by awarding one point for each correct answer, yielding a maximum total of 22 points: 12 points for correct answer to the statements regarding anaerobic activity (12a, 12b, 12c, 12d,12e, 13a, 13b, 13c, 13d,13e, 14a, 14b), 8 points for correct answer to the statements regarding aerobic activity (14c, 14d, 14e, 15a, 15b, 15c, 15d, 15e), and 1 point each for guidelines (question 11) and the frequency of prescribing physical activity in the management of diabetes (questions 17).

More than 90% correct answers indicated optimal knowledge, 71–90% correct answers indicated good knowledge, 50–70% correct answers indicated satisfactory knowledge, and less than 50% correct answers indicated insufficient knowledge. The correct answer to the instrument questions were as follows: question 11—b, question 12—a, b, d and e, question 13—a, c, d and e, question 14—c, d and e, question 15—a, c and e, and question 17—c. Adequate answer to question 18 was a, to question 19 it was c, and to question 20 it was c.

### 2.2. Conducting Survey

The next phase involved diabetologists who actively worked with persons with diabetes. A request for participation in the research was sent to professional diabetology societies from 12 countries in Southeastern Europe (Albania, Bosnia and Herzegovina, Bulgaria, Montenegro, Greece, Croatia, Kosovo, Hungary, Romania, North Macedonia, Serbia and Ukraine). Professional diabetology societies were asked to send all their specialist diabetologist members an e-mail with study information and a request to participate with a link to the instrument and an explanation that filling out the survey was anonymous and that no IP address information nor personal data were collected, in accordance with the data protection regulation. The survey was conducted from November 2022 till January 2024.

### 2.3. Statistical Analysis

Categorical data were presented using absolute numbers and percentages, and numerical variables were presented as mean ± standard deviation (SD) median and interquartile range, after testing normality of data distribution. The normality of the distributions was tested using Kolmogorov–Smirnov test, and the variance homogeneity was tested using Levene’s test. Differences in distribution of numerical variables between groups were analysed with Mann–Whitney U test. Differences in distribution of categorical variables were analysed using Pearson $x^{2}$ test.

Significance level (α) was 5% in all analyses, which were carried out using IBM SPSS Statistics for Windows, version 25 (IBM Corp., Armonk, NY, USA).

## 3. Results

**Figure 1:**
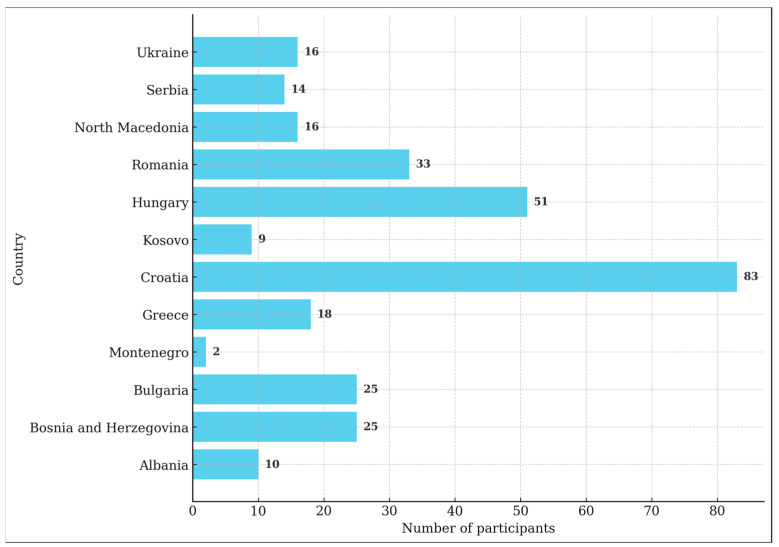
Number of participants from individual countries.

A total of 313 diabetologists participated in the research, but 11 were excluded from the analysis as they did not actively work with patients. The share of participants from individual countries is shown in Figure 1.

Characteristics of participants are shown in Table 1; 37 (12.3%) participants worked in diabetes centres, 123 (40.7%) in university hospitals, 84 (27.8%) in general hospitals, 35 (11.6%) in private healthcare institutions, and 23 (7.6%) in other institutions.

The results of the answers to all multiple-choice questions are shown in Table 2. Of all the respondents, 26.4% are familiar with recommended level of physical activity for people with diabetes. The percentage of adequate answers related to prescribing was 53.0% (question 18), 33.4% (question 19) and 25.5% (question 20), respectively.

Distribution of the knowledge scores is shown in Table 3.

The distribution of participants according to their knowledge scores is shown in Table 4.

The statement that, ideally, a physician should personally educate patients with diabetes about physical activity was found to be completely true by 25.2% of participants, while 14.6% of them completely agreed with the statement that education about the length and the type of recommended physical activity for diabetic patients should be provided by a trained professional. As many as 85.1% of the participants were personally engaged in physical activity, and 94.4% wanted to know more about the effect of physical activity on diabetes. The analysis of the entire sample showed that knowledge of the guidelines and aerobic physical activity did not influence the prescription of physical activity.

Participants were also divided into groups according to their place of work (diabetes centres and university hospitals compared to others), having a PhD, holding a teaching position, attendance at international congresses, personal engagement in physical activity and age (group ≤ 35, *n* = 68, compared to the group > 35, *n* = 234). There was no statistically significant difference in overall knowledge of the groups divided by the place of work (Mann–Whitney u test z = 0.32, *p* = 0.748), PhD (Mann–Whitney u test z = 1.01, *p* = 0.313), teaching position (Mann–Whitney u test z = 0.03, *p* = 0.968) and personal engagement in physical activity (Mann–Whitney u test z = 0.06, *p* = 0.947). In the group that attended conferences with international participation 65 of 217 participants (30%) always provide re-education on possible changes in physical activity and set new goals in case a patient’s health status changes, compared to 36 of 85 (42%), $x^{2}=4.22,\;df=1,\;P=0.039$. In the younger group 44 of 68 participants (65%) actively discuss achieved goals for physical activity and provide additional information if necessary compared to 116 of 234 (50%) in the older group ($x^{2}=4.84,\;df=1,\;P=0.028$).

## 4. Discussion

This systematic research investigated the knowledge that specialist diabetologists had about physical activity, how they recommended it to their patients, whether they were familiar with the guidelines, and how familiar they were with its importance and influence on diabetes mellitus.

### 4.1. The Knowledge on Physical Activity

Previous studies show that most physicians, including two-thirds of family doctors, half of paediatric diabetologists, and three-quarters of various specialists, are unfamiliar with physical activity guidelines [33,36,37]. The design of this study allowed for a deeper insight into knowledge about physical activity, specifically among specialist diabetologists. The results indicated satisfactory overall knowledge of physical activity. However, a large number of diabetologists did not know the basic characteristics of either aerobic or anaerobic physical activity. For example, as many as half of them were not aware of the fact that anaerobic physical activity significantly reduces HbA1c and increases the level of basal metabolism, and a third of them did not know that anaerobic physical activity has a positive effect on insulin sensitivity. Diabetologists had better results in recognising types of physical activity among the offered exercises. Their knowledge about physical activity was therefore more general, but their in-depth knowledge, although satisfactory, still needs improvement, which points to a lack of structured education, and a need for self-education on physical activity. Although nowadays quality information is more widely available than ever before, this is certainly not optimal and should by all means be improved. The optimal knowledge is a basic prerequisite for properly prescribing and transferring a positive attitude about physical activity to people with diabetes. The less-than-optimal knowledge on the other hand indicates that a significant number of people with diabetes remain deprived of adequate information about physical activity. Considering the results of previous studies, which have shown that the number of patients receiving education on physical activity is not satisfactory [38], this finding becomes even more significant. A study conducted among diabetes educators showed that a considerable number of physicians do not recommend anaerobic physical activity [39]. The results of our study indicate that diabetologists’ knowledge of anaerobic physical activity is good. But some educators may have mistaken belief that aerobic one is the only healthy physical activity, whereas anaerobic physical activity is not only undesirable but also potentially harmful. This is not true, and the risk from performing anaerobic physical activity is actually very small [40]. It is especially important to consider anaerobic physical activity if a person with diabetes cannot do aerobic exercise for any reason. Movement is rooted in the development of the human species, and most forms of physical activity are neither strictly aerobic nor anaerobic, but mixed. Such are the guidelines, which only a quarter of the participants were aware of. This percentage is similar to those found in other studies involving family physicians and specialists from various fields, indicating a general lack of emphasis on physical activity education in medical training [36,37,41]. Guidelines on physical activity should be known by every diabetologist. The effect on health of both types of physical activity is favourable and works synergistically. Although the recommendation on physical activity must be strictly individualised, given the optimal effect of this combination on glycaemic control and the development of diabetes complications, it must be an ideal that should always be strived for.

The influence of knowledge on the prescription of physical activity is not altogether simple. Data from previous studies have shown that physicians cite lack of knowledge as one of the main barriers to prescribing physical activity [33]. The results of this study confirm that their knowledge is indeed not optimal and needs to be improved. But, when looking at the entire study sample, good knowledge of the guidelines and characteristics of aerobic physical activity did not influence physicians’ prescribing habits. Therefore, further research to deepen this topic is necessary.

### 4.2. Prescription of Physical Activity

Results on prescribing physical activity showed that over 60 percent of diabetologists reported that education about physical activity should be carried out at every examination. More diabetologists expressed belief that patients should be educated about physical activity by themselves rather than by trained professionals. The proportion of diabetologists who neither personally prescribed physical activity nor provided any related education was relatively small. These results are similar to those of other studies that have shown a significant number of physicians prescribe physical activity to patients, even when their knowledge of the guidelines is not optimal [33,39]. However, a significant proportion of diabetologists only mentioned physical activity as being beneficial and did not carry out adequate education about it. Moreover, only a quarter of participants provided clear instructions in medical records. Thus, these data show that a significant number of persons with diabetes receive information about physical activity during their visit to a diabetologists office, but not in a satisfactory way. Education should not be limited to providing brief information about physical activity as merely a desirable behaviour, because patient education significantly affects the implementation of physical activity, and patients’ choice of treatment methods is most influenced by doctors’ recommendations [42]. Any improvement in the engagement of people with diabetes in physical activity is important and worth every effort. There are numerous reasons why people with diabetes are less physically active. Therefore, it is necessary to dedicate time to education; the approach must be individual and should consider limitations, comorbidities and preferences of the person with diabetes, as this increases the likelihood of success.

Younger diabetologists had better scores on prescribing physical activity. This may indicate a positive social trend that emphasises the importance of a healthy lifestyle and therefore physical activity, primarily accepted by younger people. Diabetologists who participated in congresses provided significantly less education on physical activity and set new goals in case of a change in patients’ health status in comparison with their colleagues who did not attend professional meetings. Previous studies show that most physicians receive education on physical activity primarily through conferences [33]. These results indicate the need to increase the coverage of topics related to physical activity at conferences and also highlight the need for education to be implemented at multiple levels, not solely through conferences.

It is also possible that other factors such as working conditions, i.e., the number of examinations per working day and the time available have an influence on the prescription of physical activity. A comparative analysis of diabetologists from Croatia (the only representative sample in relation to other study participants thus showed that diabetologists from Croatia had a better score on the question of recognising forms of physical activity, as there was no difference in other questions of knowledge, while in questions on prescribing, they had lower scores than the other respondents. However, they had a significantly higher number of examinations per day, and consequently less time with an individual patient, which clearly influenced the prescription of physical activity. Previous studies have also indicated a lack of time as a potential barrier to prescribing physical activity, which is confirmed by these results [33,43]. Although the number of examinations does not necessarily affect the quality of work, it leaves a doctor with less time to provide education. In these circumstances, education about pharmacotherapy obviously takes precedence over that about physical activity. The simplicity of today’s effective pharmacological solutions has probably affected not only patients who find it easier to take therapy than to change their lifestyle, but also doctors, who prescribe pharmacotherapy to achieve their goal—good glucoregulation—in a faster and easier way. Pharmacotherapy has indeed significantly improved the quality and length of life of people with diabetes. Over time, the prescription of physical activity obviously decreased, and so did related knowledge. But this is certainly inadequate for many reasons, the number of people with diabetes who do not take therapy as prescribed not being negligible and the number of those who do not reach their target glycaemic values being significant [2]. The potential of physical activity is truly significant, and it is necessary to improve the related knowledge, prescribing habits and awareness among diabetologists. Indeed, almost all participants reported wishing to know more about physical activity and its impact on diabetes. Secondly, the financial burden of diabetes, and especially its complications, to the health system is not negligible. The mere potential of physical activity in reducing HbA1c is clearly greater than that of some current pharmacological solutions. Surely one more reason for providing appropriate education on physical activity to persons with diabetes is necessary.

### 4.3. Suggestions for Improvements

The first level at which improvement is possible is education about physical activity in medical school, especially because acquiring knowledge early is important for forming a positive attitude and later prescribing habits. Previous research shows that current education in medical schools is inadequate, and that future doctors do not feel competent to prescribe physical activity to patients [44]. This can be influenced through the implementation of high-quality education. Another important opportunity to enhance knowledge and the prescribing of physical activity is a mandatory course on physical activity as part of a specialisation in diabetology. This certainly provides an opportunity for the education of future diabetologists on physical activity specifically targeted at persons with diabetes. The knowledge of diabetologists can be enhanced, and prescribing habits can be improved through continuous medical training courses organised by professional societies and held by diabetologists particularly interested in the topic, as well as physiologists, kinesiologists or sports medicine specialists. The representation of topics on physical activity in the treatment of diabetes should be increased at diabetology conferences. A positive impact can also be made by citing a concise summary of exact recommendations instead of just using the term physical activity in the guidelines for professional societies. As some recent studies show, the capabilities of health informatics in improving patient care hold great potential [45]. It is possible that informatics solutions could assist physicians in prescribing physical activity as well as in selecting the recommended type of activity based on the patient’s characteristics. Finally, further research on other possible influencing factors is needed to develop a strategy to increase the knowledge of diabetologists about physical activity and its influence on diabetes and improve prescription practices and education of persons with diabetes about the benefits of physical activity.

#### Study Limitations

The limit of this research is that the sample is not random and is only representative of Croatia. The sample from most other countries is small, which limits the ability to generalise. The data on physical activity prescription are based on self-reports, which may overestimate the actual prescribing of physical activity. When designing the instrument, the time required to complete it was taken into account, as it could not be too long in order to attract as many respondents as possible. Therefore, the amount of information collected is limited. A study that would include more characteristics of physicians, their communication skills, a more in-depth analysis of their daily practice and education implementation, as well as a broader range of physicians’ attitudes, would provide better insight into potential barriers. The vast majority (85.1%) of the diabetologists who took part in the study are physically active. It is possible that such diabetologists were more interested in participating in this research, although this could also give these results even more importance.

## 5. Conclusions

Diabetologists’ overall knowledge of physical activity was found to be at least satisfactory in 90% of participants with a median value of 15 out of 22 points. About one quarter of diabetologists are familiar with recommended level of physical activity for people with diabetes. Less than two third of diabetologists know that education on physical activity should be provided at every consultation with patients with diabetes. During the diabetic patient’s check-up, 160 (53.0%) actively discuss the achieved goals for physical activity and provide additional information if necessary; 101 (33.4%) of respondents always provide re-education on possible changes in physical activity and set new goals in case a patient’s health status changes and 77 (25.5%) provide clear instructions about the type and duration of recommended physical activity in the medical records. Prescribing often implies only informing the patient that physical activity is desirable.

## Figures and Tables

**Table 1:** Characteristics of participants.

Variables *	All (*N* = 302)
Age (in years)	47.2 ± 12.6
Female	216 (71.5%)
Number of working days in outpatient department	3.8 ± 1.4
Participants who also treat hospitalised patients	213 (70.5%)
Number of examinations during the day	16.0 ± 8.4
Duration of first examination (in minutes)	28.2 ± 12.6
Duration of control examination (in minutes)	16.6 ± 8.7
PhD	95 (31.5%)
University teaching position	105 (34.8%)
Participants who attended congresses with international participation in the preceding two years	217 (71.9%)

* continuous data are presented as mean ± SD and categorical data are presented as counts and percentages (%).

**Table 2:** Distribution of the participants according to the answers to multiple-choice questions.

Question (*N* = 302)	Answer	Correct Answer *n* (%)	Wrong Answer *n* (%)
11. Recommended level of physical activity for people with diabetes is	At least 150 min of aerobic physical activity per week in addition to at least 2 anaerobic training sessions per week	80 (26.4%)	222 (73.5)
12. Anaerobic physical activity	(a) Has a significant effect on reducing HbA1c	154 (50.9%)	148 (49.0%)
	(b) Is a good option for patients who cannot perform aerobic activities	132 (43.7%)	170 (56.3%)
	(c) Is not recommended for people with diabetes who are over 75 years old	208 (68.9%)	94(31.1%)
	(d) When combined with aerobic physical activity is the optimal form of physical activity for people with diabetes	239 (79.1%)	63 (20.9%)
	(e) For anaerobic activity, the number of exercises and their repetitions are more effective than the length of training	110 (36.4%)	192 (63.6%)
13. Anaerobic physical activity	(a) Includes high-intensity muscle workouts for a short period of time	214 (70.9%)	88 (29.1%)
	(b) Reduces HbA1c, although not clinically significantly	181 (59.9%)	121(40.1%)
	(c) Increases basal metabolic rate	158 (52.3%)	144 (47.7%)
	(d) Increases muscle mass	233 (77.2%)	69 (22.8%)
	(e) Has a positive effect on insulin sensitivity	199 (65.9%)	103 (34.1%)
14. For the most part, anaerobic exercises include	(a) Swimming	252 (83.4%)	50 (16.6%)
	(b) Dancing	264 (87.4%)	38 (12.6%)
	(c) Weightlifting	262 (86.8%)	40 (13.2%)
	(d) Push-ups	239 (79.1%)	63 (20.9%)
	(e) Sit-ups	208 (68.9%)	93 (30.8%)
15. For aerobic exercises, the following is true	(a) It is a longer physical activity with less intensity	227 (75.2%)	75 (24.8%)
	(b) Producing energy is less effective than with anaerobic exercise	259 (85.8%)	43 (14.2%)
	(c) Optimal heart rate with moderate intensity aerobic exercise is 50–70% of the maximum heart rate	228 (75.5%)	74 (24.5%)
	(d) The easiest way to establish the maximum heart rate is to subtract the patient’s age from the number 200	186 (62.0%)	116 (38.0%)
	(e) After using up glucose as a source of energy, muscles continue to use fat as a fuel	202 (66.9%)	100 (33.1%)
17. According to guidelines, education on physical activity should be provided	At every appointment	186 (61.6%)	116 (38.4)
18. During the diabetic patient’s check-up	I actively discuss achieved goals for physical activity and provide additional information if necessary	160 (53.0%)	142 (47.0%)
19. In case a patient’s health status changes	I always provide re-education on possible changes in physical activity and set new goals	101 (33.4%)	201 (66.6%)
20. In medical records (case history)	In addition to prescribing pharmacotherapy, I provide clear instructions about the type and length of recommended physical activity	77 (25.5%)	225 (74.5%)

**Table 3:** Distribution of the knowledge scores.

Variable (*N* = 302)	Total Score	Valid *N*	Median	Minimum	Maximum	Lower Quartile	Upper Quartile
Knowledge of anaerobic activity	12	302	9.0	2.0	13.0	7.0	10.0
Knowledge of aerobic activity	8	302	5.0	1.0	7.0	5.0	6.0
Overall knowledge *	22	302	15.0	3.0	22.0	13.0	17.0

* The total score also included one point each for adherence to guidelines (question 11) and for the frequency of prescribing physical activity in the management of diabetes (question 17).

**Table 4:** Distribution of the participants according to the score of knowledge (Total score = 22).

Categories (Level) of Knowledge (*N* = 302)	Number of Participants (%)
>20 (90%), optimal	12 (4%)
16–20 (71–90%), good	118 (39%)
11–15 (50–70%) satisfactory	142 (47%)
≤10 (<50%) insufficient	3 (10%)

## Data Availability

The raw data supporting the conclusions of this article will be made available by the authors on request.

## Appendix A. The Instrument

1. Your Sex: M or F		
2. Your age:____		
3. I work at:		
(a) University Hospital Centre		
(b) General hospital		
(c) Diabetes Centre		
(d) Private healthcare provider		
(e) Other		
4. How many days a week do you perform outpatient examinations: ____		
5. How many examinations do you carry out per day approximately:____		
6. Do you also treat hospitalized patients: Yes/No		
7. How long does an examination last on average: first exam: ____min, follow-ups:____min		
8. Your academic degree: MD/PhD		
9. Do you hold a university teaching position (assistant/assistant professor/professor): Yes/No		
10. Over the past 2 years, have you attended an international conference? Yes/No		
11. Recommended level of physical activity for people with diabetes is:		
(a) at least 150 min of aerobic physical activity per week		
(b) at least 150 min of aerobic physical activity per week in addition to at least 2 anaerobic training sessions per week		
(c) at least 150 min of physical activity per week regardless of the type of activity		
12. Anaerobic physical activity: (Select all that apply)		
(a) has a significant effect on reducing HbA1c		
(b) is a good option for patients who cannot perform aerobic activities		
(c) is not recommended for people with diabetes who are over 75 years old		
(d) when combined with aerobic physical activity is the optimal form of physical activity for people with diabetes		
(e) for anaerobic activity, the number of exercises and their repetitions are more effective than the length of training		
13. Anaerobic physical activity: (Select all that apply)		
(a) includes high-intensity muscle work-outs for a short period of time		
(b) reduces HbA1c, although not clinically significantly		
(c) increases basal metabolic rate		
(d) increases muscle mass		
(e) has a positive effect on insulin sensitivity		
14. For the most part, anaerobic exercises include: (Select all that apply)		
(a) swimming		
(b) dancing		
(c) weightlifting		
(d) push-ups		
(e) sit-ups		
15. For aerobic exercises, the following is true: (Select all that apply)		
(a) It is a longer physical activity with less intensity		
(b) Producing energy is less effective than with anaerobic exercise		
(c) Optimal heart rate with moderate intensity aerobic exercise is 50–70% of the maximum heart rate		
(d) The easiest way to establish the maximum heart rate is to subtract the patient’s age from the number 200		
(e) After using up glucose as a source of energy, muscles continue to use fat as a fuel.		
16. Ideally a physician should personally educate patients with diabetes about the length and type of physical exercise they should perform		
I fully disagree 1 2 3 4 5 6 7 I fully agree		
17. According to guidelines, education on physical activity should be provided:		
(a) at the first examination of a patient with diabetes		
(b) at the first examination and subsequently if needed		
(c) at every appointment		
18. During the diabetic patient’s check-up:		
(a) I actively discuss achieved goals for physical activity and provide additional information if necessary		
(b) I always stress the importance of physical activity in diabetes treatment, but I do not discuss it in detail since relevant education is provided by someone else in my team (e.g., nurse)		
(c) I personally explain and prescribe only pharmacotherapy, whereas information on physical activity is provided by someone else in my team		
19. In case a patient’s health status changes:		
(a) I always stress the importance of doing as much physical activity as possible in accordance with the person’s health status		
(b) Education and discussion on physical activity is carried out by someone else in my team (e.g., nurse)		
(c) I always provide re-education on possible changes in physical activity and set new goals		
20. In medical records (case history):		
(a) in addition to prescribing pharmacotherapy, I state that regular physical activity is required		
(b) I provide pharmacotherapy recommendations but do not mention physical activity		
(c) In addition to prescribing pharmacotherapy, I provide clear instructions about the type and length of recommended physical activity		
21. Education about the length and type of recommended physical activity for diabetic patients should be provided by a trained professional (e.g., physical therapist) and not by a diabetologist		
I fully disagree 1 2 3 4 5 6 7 I fully agree		
22. Do you engage in physical activity yourself? Yes/No		
23. Would you like to learn more about the effects of physical activity on diabetes: Yes/No		
24. Select Your country:		
(a) Albania	(e) Greece	(i) North Macedonia
(b) Bosnia and Herzegovina	(f) Hungary	(j) Romania
(c) Bulgaria	(g) Kosovo	(k) Serbia
(d) Croatia	(h) Montenegro	(l) Ukraine
